# Prevalence and predictors of penile nodules in French Guiana's sole prison facility

**DOI:** 10.1371/journal.pone.0204808

**Published:** 2018-09-27

**Authors:** Mathieu Nacher, Gulen Ayhan, Romain Arnal, Florence Huber, Celia Basurko, Agathe Pastre, Bruno Falissard, Vincent About

**Affiliations:** 1 Centre d’Investigation Clinique CIC INSERM 1424, Centre Hospitalier de Cayenne, Cayenne, Guyane Française; 2 Service de Psychiatrie, Centre Hospitalier de Cayenne, Cayenne, Guyane Française; 3 Hôpital de Jour Adultes, Centre Hospitalier de Cayenne, Cayenne, Guyane Française; 4 Unité de Consultations Ambulatoires Carcérales, Centre Hospitalier de Cayenne, Cayenne, Guyane Française; 5 CESP/INSERM U1018 (Centre de Recherche en Epidemiologie et Santé des Populations), Paris, France; Arizona State University, UNITED STATES

## Abstract

**Purpose:**

Penile implants or nodules are objects inserted beneath the skin of the penis mostly for erotic purposes. The procedure is painful and there may be complications. It is often associated with prison. Our objectives were to describe the prevalence of penile nodules among inmates in French Guiana, and to study factors associated with this practice, notably psychiatric diagnoses.

**Methods:**

The study was cross-sectional. All consenting new adult prisoners incarcerated between 01/01/2014 and 31/12/2014 at the penitentiary centre of French Guiana were included. The Mini International Neuropsychiatric Interview (MINI) was used to screen for psychiatric diagnoses.

**Results:**

Of 492, 29.6% declared having penile nodules. The median number was 4 (IQR = 2–7). The number of nodules correlated with age. There was no statistical link between the presence of penile nodules and the reasonforincarceration. Multivariate analysis showed that persons <45years with prior incarcerations, with substance addiction, and those with a history of death in the family were more likely to have penile nodules. Those with psychosis and those with suicidal risk were less likely to have penile nodules. Prisoners speaking English or Maroon languages seemed more likely to have penile implants in the multivariate model.

**Conclusions:**

Overall, 29.6% of arriving inmates had penile nodules. The practice was linked to drug addiction and was less frequent among those with psychosis and suicidal risk. Given the high HIV prevalence in prison, penile nodule may be an obstacle to condom-based prevention.

## Introduction

Penile implants or nodules are objects inserted beneath the skin of the penis.[1] Although the literature on the subject is rare, the practice has been described for a long time.[2] Thus, the Kama Sutra reported it and scientific paper was dedicated to it the first time in the 19^th^ century in Sumatra.[3]

The practice is mostly described in Asian and Slavic cultures. Self-injuries to the penis are frequent in the context of incarceration. [4,5]Since the 18^th^ century penile nodules are also strongly linked to prison, initially with the Yakuza in Japan. The most affected populations are prisoners, sailors, soldiers, and generally persons of low socioeconomic background. The objects that are inserted vary they may be marbles, pearls, plastic beads, glass or metal pellets, precious metal, silicon … These nodules have very different names in different regions: fang muk, mukhsa, or tancho balls (Thailand), persimbraons (Battak in Indonesia), bolitas (Philippines), chagan balls (Korea), grains of rice, penis marbles, or penis beading (Western Europe), pearling or sputnik(Russia), boegroe (Suriname) and its phonetic translation in French Guiana–bouglou- suggesting the regional spread of this cultural practice on the Guyana shield.[6,7] The prevalence of penile nodules is variable: [2] 1% among sailors in Indonesia; In the Philippines a study 72 of 92 men had bollitas mostly inserted following the advice of a group or a friend; 0.5% in sex clinics in Thailand; 31% of methamphetamine users in Thailand (90% had them inserted by a friend and 80% in prison); 0.64% among Russian immigrants in Israel; 22% among Yakuza inmates in Japan; 16.6% among Maroons (descendents of runaway slaves from Dutch plantations in Suriname) and 9.3% among Amerindians living on the Maroni in French Guiana.[8] A review of reports on penile nodules showed the mean age of persons was 25 years and that the average number of beads was 2.71.[2] The reported reasons why persons insert these nodules may be cultural, to show membership to a certain group, to enhance sexual pleasure of the sex partners, or on the contrary to inflict pain during intercourse. Insertion of such object beneath the foreskin may lead to complications ranging from pain, inflammation, hemorrhage, infections including gangrene. They may also be associated with increased risk of sexually transmitted infections, condom rupture and sexual partner injury.[2] In the context of French Guiana, where HIV infection is prevalent[9], notably among inmates, where it is associated with poor outcomes[10], penile nodules may thus present an additional risk. In addition, they are a frequent cause of emergency calls for interventions ranging from suturing at the prisons medical facility to medical evacuation when deep vascular lesions and uncontrolled bleeding require surgical exploration.

French Guiana is a French overseas territory located between Brazil and Suriname. It has the highest GDP per capita in Latin America and thus attracts numerous migrants in search of a better life. Single parent families represent a quarter of families, and 20% of families have four children or more, conditions that further increase social vulnerability[11]. The gold in the soil attracts numerous illegal gold miners. French Guiana is also a major passage for cocaine trafficking towards Europe. Drugs are cheap and there are high rates of substance use[12]. French Guiana has the highest incarceration rate (328 per 100 000) among French territories, a rate that is higher than that of Brazil (319 per 100 000), Colombia (231 per 100 000) or Venezuela (173 per 100 000).[13] There is only one correctional facility in French Guiana. It is saturated and its overpopulation and poor living conditions have been repeatedly pointed out.[14] Mental health problems are more frequent in prisoners than in the general population [15–17]. French Guiana is a land of immigration and it is thus composed of a very diverse population. This diversity of customs and languages is also found among inmates. Until recently, there had never been any study of the mental health issues of detainees in this particular territory at the crossroad between France and Latin America. Our objectives were therefore to describe the prevalence of penile nodules among inmates in the sole prison of French Guiana, and to study factors associated with this practice, notably psychiatric diagnoses.

## Methods

The study was cross-sectional. All consenting new adult prisoners incarcerated between 01/01/2014 and 31/12/2014 at the penitentiary centre of French Guiana were included. Inmates with legal guardianship were excluded so not to compromise the procedures for our study.

After incarceration, new inmates are seen by a doctor of the “Unité de consultation et de soins ambulatoires (UCSA) “, the ambulatory care unit of the prison and then by a psychiatrist or psychiatric nurse in the “Unité fonctionnelle de psychiatrie intra-carcérale (UFPI)“, the psychiatric ward. Patients could be included up to 15 day after arrival. In addition to this normal procedure we added the Mini International Neuropsychiatric Interview (MINI). The MINI is a short diagnostic structured interview (DSI) developed in France and in the United States that explores 17 disorders according to the diagnostic criteria of the Diagnostic and Statistical Manual (DSM)-IV. The reliability and validity of the MINI has been confirmed in several authors[18,19]. It has been translated and validated in 46 languages. The MINI is structured in order to allow use by non-specialized interviewers for the research of current disorders. It is among the most used psychiatric diagnostic tools[20]. For each diagnosis, one or two screening questions rule out the diagnosis if answered negatively. The MINI is therefore adapted for epidemiological studies that require a short and robust tool. The estimated duration of the interview is 15 minutes. The MINI has been translated and validated in major languages found in French Guiana: French, English, Portuguese, Dutch and Spanish. All psychiatrists and nurses using the MINI were trained in order to use the questionnaire correctly. Other questions were added: Socio-demographic variables (age, birth place, residence, languages, presence of a translator, family status, children, siblings, position among siblings, professional situation), detention history (reason for detention, prior imprisonment), psychiatric history. Detainees were also asked if they had penile nodules (“bouglous”) and how many units. Before the study started, a training period tested the feasibility of the MINI and familiarized staff with the study protocol and ensured that the staff mastered the tool before starting the inclusions.

### Inclusion criteria

Incarcerated adults accepting to participate were included.

### Exclusion criteria

Minors, persons requiring a legal guardian, or persons declining to participate were not included.

### Statistical analysis

The descriptive analysis of qualitative and quantitative variables, was followed by a bivariate analysis to identify significant variables associated with penile nodules. The variables were included in a multivariate logistic regression model to identify independent diagnoses associated with penile nodules. The modelling strategy was exploratory and the diagnostic variables were retained in the model except obsessive compulsive disorder, anorexia and bulimia which were too rare to be included in a multivariate model (15, 5 and 3 persons, respectively). Colinearity was ruled out using the collin package (STATA, College Station, Texas) and ensuring that variance inflation factors were <4. The Hosmer-Lemeshow goodness-of-fit test was used to test the model.

A similar procedure was used for bivariate and multivariate analyses of explanatory variables associated with repeated incarcerations.

Principal component analysis was performed and a loading plot of the different diagnoses was performed in order to look for potential clusters of variables.

Stata13 (College Station, Texas, USA) was used for the analysis.

### Ethical and regulatory aspects

Given the high prevalence of mental illness, the study of mental illness in prison in French Guiana was a consensual topic for health practitioners, psychiatrists, and prison administrators.[17] In addition, the study protocol was submitted to the regulatory authorities and institutional regulatory board. The study was approved by the Comité de Protection des Personnes, (CPP) of Bordeaux which was the reference for overseas territories (reference number DC 2012/115). In addition, the study was approved by the Ethical committee of INSERM CEEI in 2013 (IRB00003888). Study participation was voluntary. Persons were informed that they were free to refuse or withdraw without any negative consequences. Inmates gave oral and written informed consent to participate in the study.

## Results

### General results

**Fig 1:**
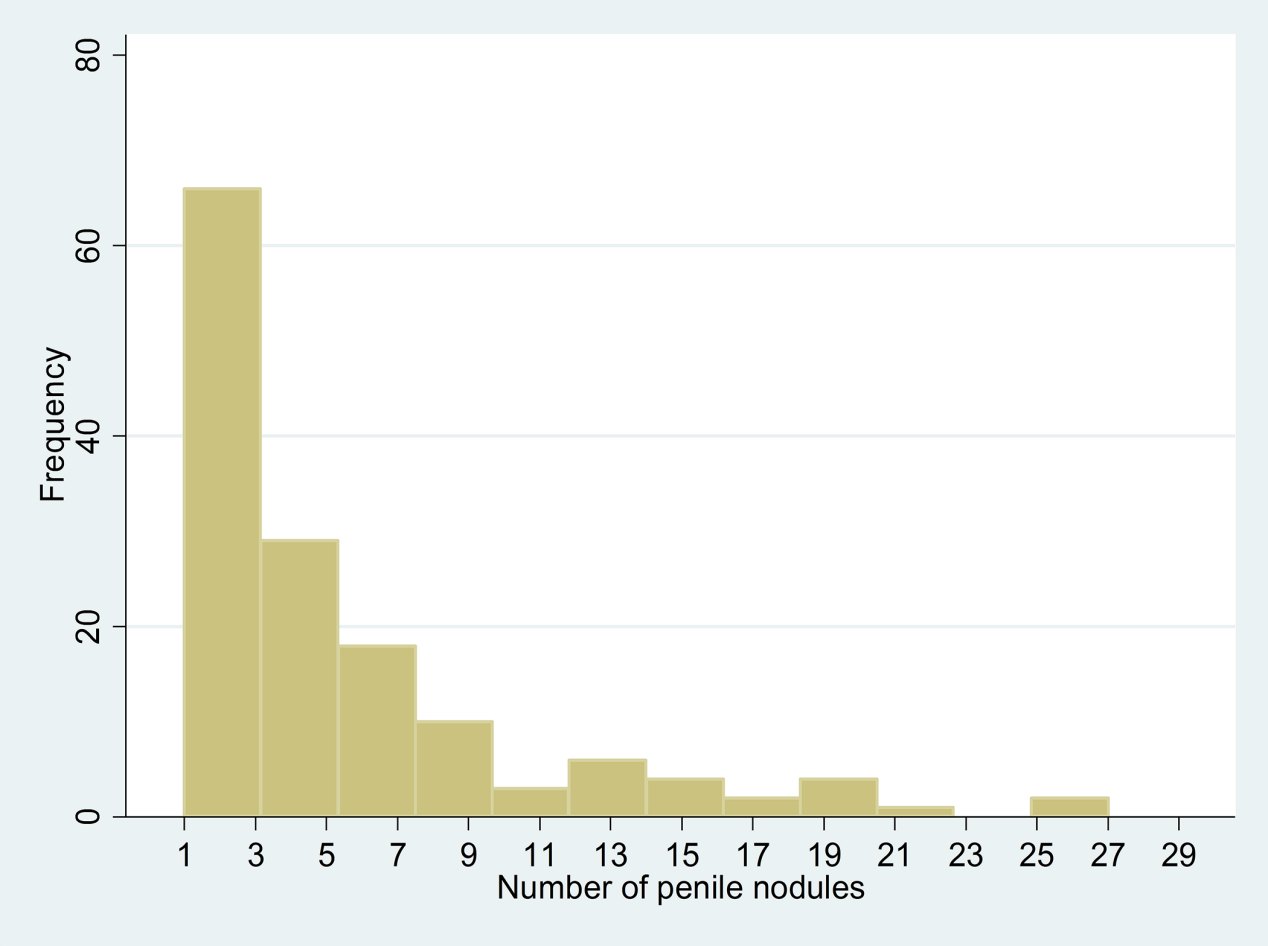
Distribution of the number of penile nodules among inmates in French Guiana.

The data set had no missing values. Of the 492 included male detainees, 29.6% declared they had penile nodules. Among those with penile nodules, the median number was 4 (IQR = 2–7). Fig 1 shows the distribution of the number of penile nodules. Among those who had a penile nodule, the number of nodules was correlated with age (rho = 0.18, P = 0.02). Among 7 HIV-infected patients surveyed, 4 had penile nodules.

**Fig 2:**
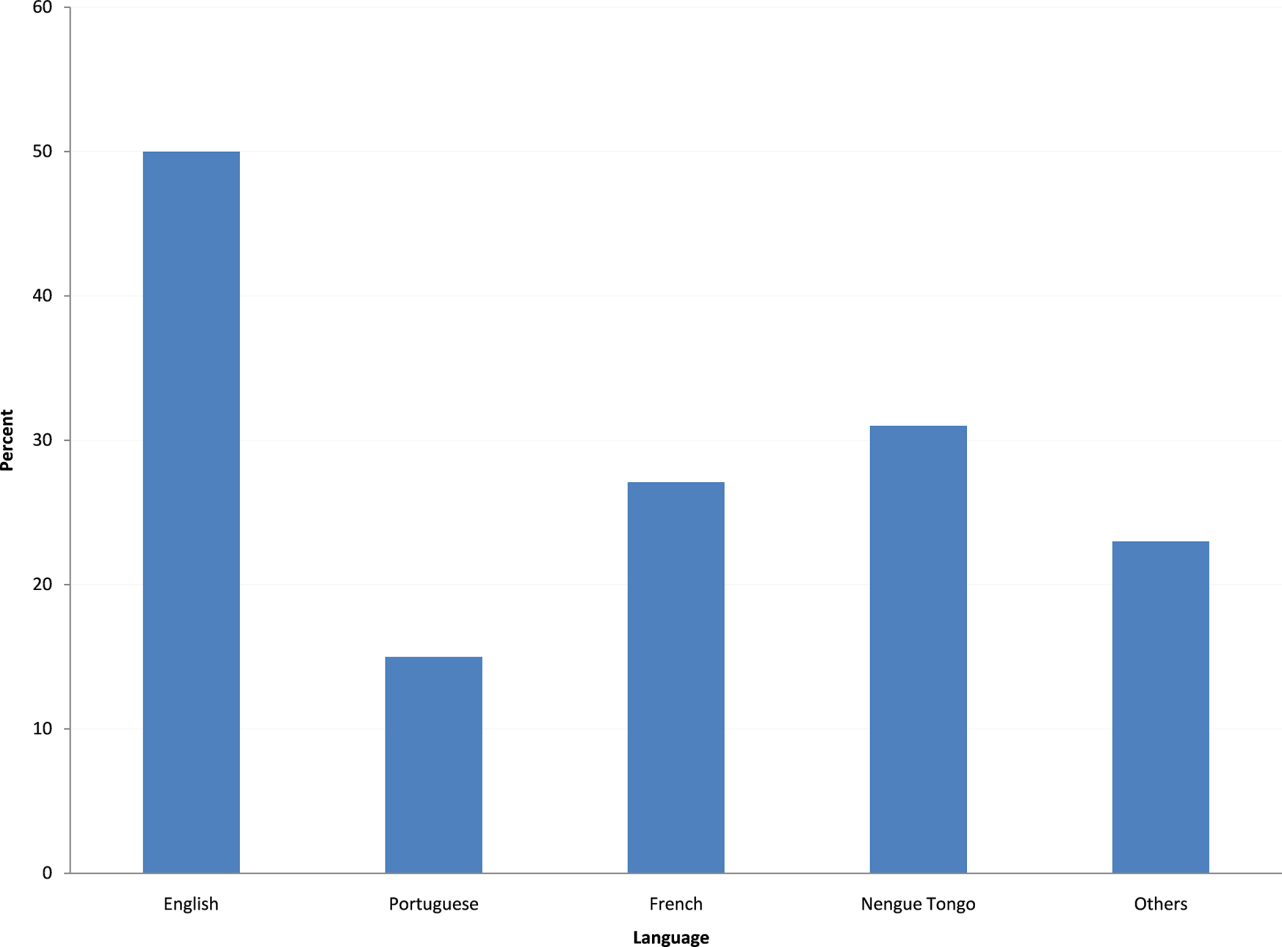
Proportion of male inmates with penile nodules, by language spoken.

French language and creole are the main languages spoken in the general population. There were differences by language spoken with persons speaking English (mostly from Guyana)and those speaking maroon languages (Sranan tongo, nenege tongo, Saamaka, aluku, ndjuka) having a greater “prevalence” of penile nodules (Fig 2).

### Penile nodules and prior incarcerations

When looking at the relation between the presence of penile nodules and the number of incarcerations there was a significant linear trend (P = 0.01).The prevalence of penile nodules in those who had never been incarcerated before was 41/252 (16%) versus 104/240 (43%).

### Penile nodules and causeof incarceration

There was no statistical link between the presence of penile nodules and the cause of incarceration (Chi2 (df = 10) = 10.3, P = 0.58). Notably, rape as a cause of incarceration was not significantly associated with penile nodules (22.7% when rape was the cause of incarceration) (29.8% when rape was not mentioned, p = 0.47). When recoding the causes of incarceration by violent versus non violent there was no difference (29.2% *vs* 29.6%, respectively, P = 0.92).

### Penile nodules and mental illness

**Fig 3:**
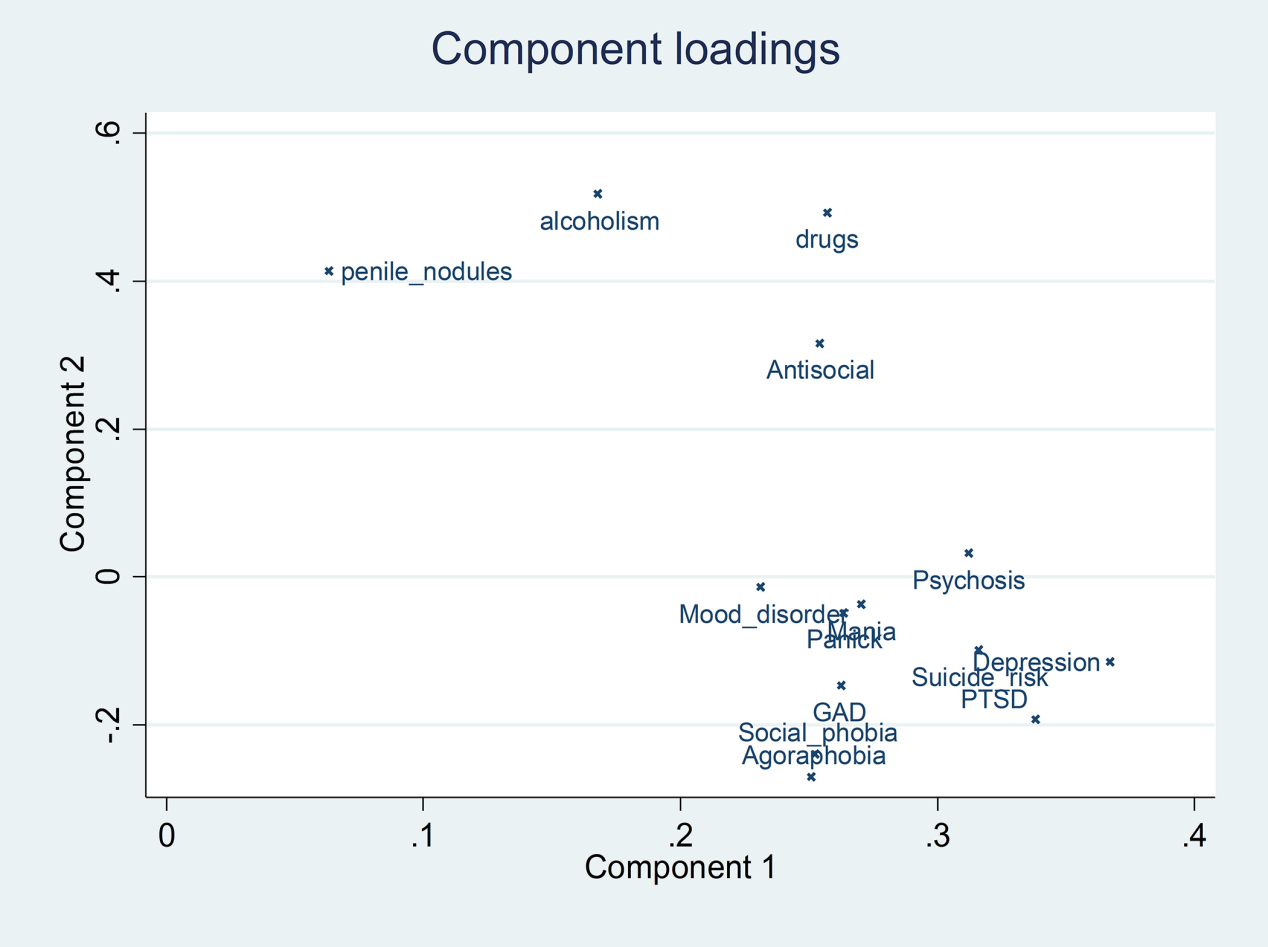
Principal component analysis showing the component loadings for psychiatric diagnoses and the presence of penile nodules.

When looking at any psychiatric disease persons with any psychiatric diagnosis were more likely to have penile nodules OR = 2.5 (95%CI = 1.5–4.3), P = 0.002 (34.3% of those with any psychiatric diagnosis and 17.2% of those without any). Table 1 shows the detail of the link between penile nodules and different psychiatric diagnoses. Overall, the multivariate analysis showed that persons <45 with prior incarcerations, with substance addiction, and those with a history of death in the family were more likely to have penile nodules. On the contrary, those with psychosis and those with suicidal risk were less likely to have penile nodules. Although, not statistically significant at the 5% level, prisoners speaking English or Maroon languages (sranan tongo, nenege tongo, saamaka, aluku, or ndjuka) seemed more likely to have penile implants in the multivariate model. The test for goodness of fit was satisfactory (P = 0.7). Fig 3 shows the loading plots after the principal component analysis of penile nodules and psychiatric diagnoses.

**Table 1:** Variables associated with the presence of penile nodules in arriving inmates in French Guiana.

	Bouglous N(%)	No Bouglous N(%)	Crude odds ratio (95% CI)	Adjusted odds ratio (95% CI), P
***Age***				
**18–25**	60 (27.7)	160 (72.7)	1.4 (0.9–2.9)	3.2(1.2–8.1), P = 0.01
**26–35**	46 (33.1)	93 (66.9)	1.9 (0.9–3.9)	2.6 (1.02–6.5), P = 0.04
**36–45**	27 (36)	48 (64)	2.1(0.98–4.7)	2.8 (1.08–7.4), P = 0.03
**46–71**	12 (20.7)	46 (79.3)	1	1
***Prior incarceration***				
***Yes***	104 (43.3)	136(56.7)	3.9 (2.6–6)	4 (2.4–6.7), P<0.001
***No***	41 (16.3)	211 (83.7)		
***Language spoken***				
***English***	32(50)	32(50)	2.7(1.5–4.7)	1.9 (0.96–6.5), P = 0.06
***Portuguese***	6(15)	34(85)	0.5(0.2–1.2)	0.4 (0.1–1.3), P = 0.14
Maroon language**	22(31)	49(69)	1.2(0.7–2.1)	1.8(0.94–3.5), P = 0.07
***French***	79(27.1)	212(72.8)	1	1
***Others***	6(23)	20(77)	1.2(0.5–3.2)	0.7(0.1–4.1), P = 0.6
***Antisocial disorder***				
**Yes**	65(38.4)	104 (61.6)	1.9 (1.3–2.8)	1.4 (0.86–2.3), P = 0.17
**No**	80 (24.8)	243(75.2)		
***Generalized anxiety disorder***				
**Yes**				
**No**	39(32)	83(68)	1.2 (0.7–1.8)	1 (0.6–1.7), P = 0.9
	106(28.7)	264(71.3)		
***Psychosis***				
**Yes**	6(16.2)	31(83.8)	0.4 (0.17–1.1)	0.1(0.05–0.5), P = 0.002
**No**	139(30.6)	316(69.4)		
***Substance addiction***				
**Yes**	74(45.4)	89(54.6)	3(2–4.5)	2.9 (1.7–4.7), P<0.001
**No**	71(21.6)	258(78.4)		
***Alcohol addiction***				
**Yes**	37(41.6)	52(58.4)	1.2 (0.8–1.8)	1.1 (0.6–2), P = 0.7
**No**	108(26.8)	295(73.2)		
***PTSD***				
**Yes**	26(34.2)	50(65.8)	1.3(0.8–2.2)	1.5 (0.7–2.9), P = 0.2
**No**	119(28.6)	297(71.4)		
***Social phobia***				
**Yes**	13(31)	29(69)	1.1 (0.5–2.1)	1.1 (0.4–2.6), P = 0.8
**No**	132(29.3)	318(70.7)		
***Agoraphobia***				
**Yes**	17(31.5)	37(68.5)	1.1 (0.6–2)	1 (0.4–2.2), P = 0.9
**No**	128(29.2)	310(70.8)		
***Panic attacks***				
**Yes**	9(26.5)	25(73.5)	0.8 (0.4–1.9)	0.7 (0.1.9), P = 0.5
**No**	136(29.7)	322(70.3)		
***Mania***				
**Yes**	2(13)	13(86.7)	0.4 (0.1–1.6)	0.2 (0.03–1.1), P = 0.07
**No**	143(30)	334(70)		
***Mood disorders***				
**Yes**	4(25.3)	13(76.5)	0.7 (0.2–2.3)	0.9 (0.2–4.1), P = 0.9
**No**	141(29.7)	334(70.3)		
				
***Suicidal risk***				
**Yes**	16(27.1)	43(72.9)	0.9 (0.5–1.6)	0.6 (0.2–1.4), P = 0.02
**No**	129(29.8)	304(70.2)		
***Depression***				
**Yes**	18(32.14)	38 (67.9)	1.1 (0.6–2.1)	1.1 (0.5–2.5), P = 0.7
**No**	127(29.1)	309(70.9)		
History of death in the family*				
**Yes**	60(36.4)	105 (63.6)	1.6 (1.1–2.4)	2 (1.2–3.3), P = 0.009
**No**	82(25.9)	235(74.1)		

*(141) 85% reported the death of a parent (father/mother), 11(6.6%) the death of a brother or sister, 6 the death of grandparents, 2 the death of a child, 2 the death of a spouse, 1 the death of an uncle/aunt, and 2 the death of other family relationships.

** Sranan tongo, nenegetongo, saamaka, aluku, or ndjuka.

## Discussion

Overall 29.6% of the respondents in the Remire-Montjoly Penitentiary declared having penile nodules. The practice seemed more frequent in those who had a history of prior incarceration, who were younger than 45 years, those with a history of drug addiction variables which are in accordance with the published literature[1,2]. It is noteworthy that each of these variables was independently associated with penile nodules. Penile nodules are frequently inserted during imprisonment but they are also commonly inserted outside of prison too, for a fee, by persons that are not health professionals and have no legal authorization to conduct these invasive procedures. Hence 16% of the surveyed population who had never been imprisoned before had penile nodules. There was also an independent statistically significant association between the presence of penile nodules anda history of death in the family. To our knowledge this had never been reported in the literature. The intended “erotic” function of penile nodules, in the context of frequent sexual deprivation of inmates is a frequent explanation, the ritual display of courage and belonging to a group is another frequent interpretation.However, we have no straightforward explanation for the association with death of a family member, perhaps further anthropological studies may enlighten us on some of the motivations for inserting penile nodules in our region. Finally, there was a negative association between penile nodules, and psychosis and suicide risk.Perhaps these mental states change the salience of the sexual and/or social motivations for inserting penile nodules. Again this would require qualitative analyzes to better understand these statistical associations.

The HIV prevalence in prison oscillates between 4 and 5% in French Guiana. There is a very high mortality among HIV-patients discharged from prison[10]. If 29.6% of detainees have penile nodules this implies that condom use will be difficult and therefore there will be an increased risk for acquiring and transmitting HIV. This is even more likely when factoring in the substance addiction problem[21], the interruption of antiretroviral treatments by HIV-infected patients. Here, there seemed to be a greater proportion of persons with penile nodules in HIV-infected inmates than in HIV-negative inmates, but this did not reach statistical significance perhaps because the number of HIV-infected patients surveyed was small.

There are several limitations to our study: The cross sectional design and the use of self-reported data in a context of incarceration may have led to biased estimates. The MINI scale is a validated screening tool but it may not always be sufficiently sensitive notably for Attention deficit disorders and certain borderline personality disorders which are associated with incarceration. The inclusion of study participants right after incarceration–a stressful period- may have impacted responses to the questions. Finally, persons were interviewed on arrival, and a number of persons insert penile nodules during their incarceration. Since half of the persons interrogated had never been to prison before, it is thus likely that the prevalence would be higher if we had surveyed persons leaving prison or persons in prison for a longer period of time. Despite these limitations, the acceptance rate was good and the sample size was significant thus represents in our view a valid cross section of inmates in French Guiana.

In conclusion, the present study shows that 29.6% of arriving inmates have penile implants and that the frequency of this adornment is higher in those with a history of incarceration and drug addiction.A family history of death was associated with penile nodules though we did not explore this further. Psychoses and suicide risk were negatively associated with penile nodules. In a context of high HIV prevalence, this very common practice combined the with behavioral and social consequences of substance abuse may significantly hamper primary and tertiary prevention measures and may thus fuel the HIV epidemic[17, 21].
